# Multi-Omics Analysis Reveals a Regulatory Network of ZmCCT During Maize Resistance to Gibberella Stalk Rot at the Early Stage

**DOI:** 10.3389/fpls.2022.917493

**Published:** 2022-06-23

**Authors:** Bozeng Tang, Zhaoheng Zhang, Xinyu Zhao, Yang Xu, Li Wang, Xiao-Lin Chen, Weixiang Wang

**Affiliations:** ^1^Beijing Key Laboratory of New Technology in Agricultural Application, National Demonstration Center for Experimental Plant Production Education, College of Plant Science and Technology, Beijing University of Agriculture, Beijing, China; ^2^State Key Laboratory of Agricultural Microbiology and Provincial Hubei Key Laboratory of Plant Pathology, College of Plant Science and Technology, Huazhong Agricultural University, Wuhan, China

**Keywords:** multi-omics, gibberella stalk rot, *Fusarium graminearum*, plant resistance, transcriptomics, metabolomics, maize disease

## Abstract

Gibberella stalk rot (GSR) caused by *Fusarium graminearum* is one of the most devastating diseases in maize; however, the regulatory mechanism of resistance to GSR remains largely unknown. We performed a comparative multi-omics analysis to reveal the early-stage resistance of maize to GSR. We inoculated *F. graminearum* to the roots of susceptible (Y331) and resistant (Y331-ΔTE) near-isogenic lines containing GSR-resistant gene *ZmCCT* for multi-omics analysis. Transcriptome detected a rapid reaction that confers resistance at 1–3 hpi as pattern-triggered immunity (PTI) response to GSR. Many key properties were involved in GSR resistance, including genes in photoperiod and hormone pathways of salicylic acid and auxin. The activation of programmed cell death-related genes and a number of metabolic pathways at 6 hpi might be important to prevent further colonization. This is consistent with an integrative analysis of transcriptomics and proteomics that resistant-mediated gene expression reprogramming exhibited a dynamic pattern from 3 to 6 hpi. Further metabolomics analysis revealed that the amount of many chemical compounds was altered in pathways associated with the phenylpropanoid biosynthesis and the phenylalanine metabolism, which may play key roles to confer the GSR resistance. Taken together, we generated a valuable resource to interpret the defense mechanism during early GSR resistance.

## Highlights

- Through a multi-omics strategy, this study comprehensively revealed ZmCCT-associated maize defense response to Gibberella stalk rot at the early stage of *Fusarium graminearum* infection.

## Introduction

Gibberella stalk rot (GSR), one of the maize stalk rot diseases, is a severe soil-borne disease in maize (*Zea mays* L.), which has become a major threat to maize production (Jackson-Ziems et al., 2014). GSR is caused by the fungus *Fusarium graminearum*, which severely reduces both the yield and the quality of maize and produces various mycotoxins (Ledencan et al., 2003). In addition to crown rot, root rot, and seedling blight, Fusarium Head Blight is well-known to be caused by *F. graminearum* (Stephens et al., 2008; Wang et al., 2015). As a hemibiotrophic fungal pathogen, *F. graminearum* undergoes a biotrophy phase at the early stage and a necrotrophy phase at the later stage (Glazebrook, 2005). During infection, the hyphae of *F. graminearum* emerge intercellularly at the early stage (before 24 h post-inoculation, hpi), emerge intracellularly and intercellularly in the middle stage (36–48 hpi), and grow rapidly at the later stage (after 72 hpi), when the plant cell structure collapsed and tissue rotted (Glazebrook, 2005; Brown et al., 2010; Kazan et al., 2012).

Plants have evolved a repertoire of molecules to facilitate response to biotic stress mainly via a two-layer innate immunity by detecting pathogen-derived molecules, including pattern-triggered immunity (PTI) and effector-triggered immunity (ETI) (Jones and Dangl, 2006; Ngou et al., 2022). Some studies have focused on plants responding to *F. graminearum* infection, including wheat, barley, and maize. It has been reported that defense-related hormones, reactive oxygen species (ROS), pathogenesis-related (PR) proteins, and cellular detoxification-related proteins are used by plants to restrict *F. graminearum* infection (Dodds and Rathjen, 2010; Ding et al., 2011; Chetouhi et al., 2016). However, the underlying mechanisms of how maize resists *F. graminearum* at different infection stages are also limited. Therefore, it is necessary to make full use of the central resistance genes to understand the regulation mechanism of maize at different *F. graminearum* infection stages. In the past decade, comparative transcriptomic analyses of near-isogenic lines (NILs) that differ by the presence or absence of resistance-related loci have revealed insights into the potential mechanisms of defense response to *F. graminearum* in wheat or maize (Yang et al., 2010; Zhuang et al., 2013; Huang et al., 2016; Liu Y. et al., 2016). These studies identified numerous differentially expressed genes (DEGs) between GSR-resistant (R) and -susceptible (S) maize genotypes to dissect the molecular mechanisms of maize–*Fusarium* interactions by discovering key genes. The single transcriptomic analysis provides a landscape of defense response at a given time point (Ye et al., 2013); however, not all transcriptional changes observed are translated into proteins or metabolites. Therefore, combining transcriptomic data with those proteomic and metabolomic approaches can be used to comprehensively reveal the multilevel landscape of plant defense response and better understand the molecular mechanism of GSR resistance (Zhou et al., 2019; Sun et al., 2021). Recently, an integrative analysis combining transcriptomics and metabolomics showed that *ZmHIR3* controls cell death at the later stage of GSR, probably through the transcriptional regulation of key genes and functional metabolites (Sun et al., 2021).

So far, there are only several genes in maize that provide resistance to GSR, and due to the soil-borne infection pattern of *F. graminearum*, it is hard to effectively control GSR. Therefore, cloning and utilization of the GSR resistance genes became an effective strategy in controlling GSR (Yang et al., 2010; Zhang X. W. et al., 2012; Ye et al., 2013; Ma et al., 2017). The GSR resistance is controlled by quantitative resistance genes, which involve complex regulatory mechanisms. Several quantitative trait loci (QTLs) and relevant genes for GSR resistance were identified in the past two decades, including qRfg1, qRfg2, and qRfg3 (Yang et al., 2010; Zhang D. et al., 2012; Ye et al., 2013; Ma et al., 2017). Among these, qRfg1 is a major GSR resistance locus, and the GSR resistance gene *ZmCCT* was cloned from this locus (Yang et al., 2010; Wang et al., 2017). Although several GSR resistance loci and genes have been reported, the regulatory mechanisms of GSR resistance remain largely unknown. Therefore, it is necessary to make full use of GSR resistance genes to understand the regulation mechanism of maize, especially the key genes, pathways, and regulatory networks at different *F. graminearum* infection stages.

*ZmCCT* plays a dual function in photoperiod sensitivity and GSR resistance. Compared with the temperate maize, *ZmCCT* expresses much higher in teosinte maize and is responsible for delayed flowering under long days conditions (Yang et al., 2010; Hung et al., 2012). *ZmCCT* plays its role in a tissue-specific pattern that shows strong photoperiod sensitivity in leaves but conferred a stable defense response in roots during *F. graminearum* infection (Wang et al., 2017). This *ZmCCT*-dependent photoperiod sensitivity and GSR resistance could be separated by modifying a polymorphic CACTA-like transposable element (TE1) in its promoter region. The TE1 element in the promoter region of *ZmCCT* dramatically reduced maize flowering time (Yang et al., 2013). It was found that both repressive H3K27me3/H3K9me3 and active H3K4me3 histone marks enriched the non-TE1 *ZmCCT* allele. Upon *F. graminearum* infection, the non-TE1 *ZmCCT* allele functioned as a major epigenetic factor in the regulation of *ZmCCT* expression by a rapid reduction in the H3K27me3/H3K9me3 level and a progressive decrease in H3K4me3 level, which conferred to the GSR resistance (Wang et al., 2017). However, the downstream regulatory mechanisms of *ZmCCT* in photoperiod sensitivity and GSR resistance are still largely unknown.

In this study, we set out to reveal regulatory mechanisms of maize resistance to GSR at the early stage of *F. graminearum* infection. We utilized maize near-isogenic lines, Y331 and Y331-ΔTE, for testing. In Y331, a TE1 element located at the upstream sequence of *ZmCCT* promoter leads to a compromised expression of *ZmCCT* and confers susceptibility to GSR, while in Y331-ΔTE, the TE1 element was removed and confers resistance to GSR. The expression of *ZmCCT* in Y331-ΔTE is transcriptionally induced by *F. graminearum* at 1 to 3 hpi and reduced quickly, suggesting that it plays key roles at the early stage during *F. graminearum* infection. A comparative multi-omics analysis was then performed by using Y331 and Y331-ΔTE to generate RNA-seq, proteomic, and metabolomic data after inoculation with *F. graminearum* at different time points. The integrative analysis of multi-omics data demonstrated a regulatory network of defense response at the early stage of GSR.

## Materials and Methods

### Plant Materials

The maize (*Zea mays* L.) susceptible near-isogenic line Y331 and resistant near-isogenic line Y331-ΔTE are used as published previously (Wang et al., 2017). Y331 contains a susceptible ZmCCT allele with the insertion of transposon TE in the promoter region. Y331-ΔTE carries a disease-resistant ZmCCT allele which does not contain the transposon insertion.

### Inoculation of *Fusarium graminearum*

*Fusarium graminearum* was cultured at 25°C for 5∼7 days for propagation. The culture medium was cut into small pieces and put into a mung bean soup culture medium. The fungal strain was incubated in dark at 28°C and 200 rpm for 10 days, then the spores were harvested with a double-layer gauze, and the spore concentration was adjusted to 10^7^ spores/ml. The root of 2-week-old (one-leaf period) maize seedlings with spore suspension was immersed, shaken for 1 h at 28°C and 200 rpm, and then placed into an incubator to observe the disease state of maize. The root samples infected with *F. graminearum* were collected at different time points (0, 1, 3, 6 or 24 hpi) for RNA-seq, proteomic, and metabolomic analysis.

### Microscopic Observation of *Fusarium graminearum* Infection

For visualization of the growth of *F. graminearum* in maize root, the WGA-Alexa Fluor 488 dye (Thermo Fisher Scientific, Waltham, MA, United States) was used to stain the infection hyphae of the fungus. At different time points, the *F. graminearum*-infected maize roots were harvested and gently washed with distilled water, treated with 50% ethanol for 4 h and with 20% KOH at 85°C for 1.5 hs, rinsed thoroughly with distilled water and PBS buffer, and then stained with WGA-Alexa Fluor 488 (1 μg/ml) for 10 min. The stained samples were used to observe the *F. graminearum* infection process under a confocal microscope Leica TCS SP8 (Leica Microsystems, Mannheim, Baden-Württemberg, Germany).

### RNA-Seq Analysis

The maize root samples (Y331 and Y331-ΔTE, 2-week-old seedlings) inoculated with *F. graminearum* were collected at 0, 1, 3, and 6 hpi. Three biological replicates were used for assessment. Total RNA from the maize root samples was extracted using TRIzol reagent (Invitrogen, Carlsbad, CA, United States). The mRNA was enriched and purified using poly-T oligo-attached magnetic beads. Then, under elevated temperature, the purified mRNA was fragmented into small pieces using divalent cations. The RNA fragment was copied into the first-strand cDNA using reverse transcriptase and random primers. The second strand cDNA was then synthesized using DNA polymerase I and RNase H. Then, these cDNA fragments added a single “A” base and were connected to the adapter. The product was purified and enriched by PCR amplification. Subsequently, we quantified the PCR yield by a qubit and gathered the samples together to form a single-stranded DNA cycle (ssDNA cycle) so as to obtain the final library. DNA nanoballs (DNB) were generated by single-stranded DNA rings during sequencing by rolling circle replication (RCR) to amplify fluorescence signals. DNB was loaded into the patterned nanoarray, and a 100-bp opposite end reading was taken on the MGI2000 platform for the following data analysis. The MGI2000 platform combines DNA nanosphere-based nanoarrays and step-by-step sequencing using combined probe anchor synthesis sequencing methods (Beijing Genomics Institution, Bejing, China).

The raw reads were assessed using FastQC and trimmed using trimmomatic (Bolger et al., 2014). Using Hisat2 (v.2.2.0) (Kim et al., 2015), the cleaned reads were aligned against the B73 maize reference genome (Ensembl Genomes database) (Schnable et al., 2009). Differentially expressed genes were analyzed by DESeq2 (Love et al., 2014), and the expression with log2| FC| > 1 with less than 0.05 *p*-adjust values was defined as DEGs. Gene Ontology enrichment analysis was performed by AgriGO toolkits (Du et al., 2010) using p-adjust value cutoffs (0.05) for significances. KEGG enrichment of metabolic pathways and Gene Ontology term analysis were analyzed by the R package clusterProfilter v3.16 (Yu et al., 2012). Pathview (Version 3.11) was used to produce detailed mapping for the selected pathways (Luo et al., 2017). For the weighted correlation network analysis (WGCNA) analysis, only genes with at least five reads aligned to the maize genome from all the samples were kept. A total of 24,894 genes were processed as input by WGCNA (Langfelder and Horvath, 2008). The TMM values were obtained after normalization by using raw read counts. The function blockwiseModules was used to produce a network of Pearson’s correlation matrix to examine the similarity between genes. A soft power threshold of 12 was chosen because it was the lowest power to obtain the lowest correlation value (0.9) from topology analysis. Module detection was generated by modified settings to minimize the numbers of clusters by using min Modules Size = 100, merge Cut Height = 0.30. For each module, the expression of the hub gene which represents the expression of each module was generated by choosing TopHubInEachModule function.

### Data Independent Acquisition Proteomic Analysis

Maize root samples (Y331 and Y331-ΔTE) inoculated with *F. graminearum* were collected at 0, 1, 3, and 6 hpi. Three biological replicates were used for assessment. The total protein was then extracted, 5 times the volume of acetone was added, and placed at –20°C for 2 h. Then, the samples were centrifuged at 25,000 g at 4°C for 20 min, and the supernatant was separated. The resulting particles were air-dried and then 200 μL buffer (7 M urea, 2 m thiourea and 20 mm Tris HCl, pH 8.0) was added to redissolve the particles. Subsequently, 20 μL of 10 mM dithiothreitol was added at 56°C for 1 h, and then, 20 μL of 55 mM iodoacetamide was added for alkylation in the dark at room temperature for 45 min, followed by centrifugation at 4°C and 25,000 *g* for 20 min. The supernatant of protein concentration was determined using Bradford Protein Analytical Kit (Bio-Rad, Hercules, CA, United States). Around 100 μg of proteins was digested with trypsin using an enzyme-to-protein ratio of 1:40 (v/v). Then, the mixture was incubated at 37°C for 12 h. The peptides were subsequently hydrolyzed and desalted by a Strata X column (Waters, Milford, MA, United States). The peptide solution was lyophilized, and the toxic dissolved peptide was placed at –20°C for use. The sample protein concentration was calculated based on the standard curve using the Bradford quantification method. Every 10 μg of protein solution was mixed with an appropriate amount of loading buffer, heated at 95°C for 5 min, and centrifuged at 25,000 *g* for 5 min, and the supernatant was poured into a well of a 12% SDS polyacrylamide gel. After electrophoresis, the gel was stained with Coomassie brilliant blue and then decolored with a decoloring solution (40% ethanol and 10% acetic acid). For protein enzymatic hydrolysis, 100 μg of protein solution was taken per sample and diluted with 50 mM NH_4_HCO_3_ by four times the volume. Then, 2.5 μg of Trypsin enzyme was added in the ratio of protein, enzyme = 40:1, and digested for 4 h at 37°C. Trypsin was added one more time in the above ratio and digested for 8 h at 37°C. Enzymatic peptides were desalted using a Strata X column and vacuumed to dryness. For high pH RP separation, all samples were taken at 10 μg, respectively, to mix, and 200 μg mixture was diluted with 2 mL of the mobile phase A (5% ACN pH 9.8) and injected. The Shimadzu LC-20AB HPLC system coupled with a Gemini high pH C18 column (5 μm, 4.6 × 250 mm) was used. The sample was subjected to the column and then eluted at a flow rate of 1 mL/min by gradient: 5% mobile phase B (95% CAN, pH 9.8) for 10 min, 5% to 35% mobile phase B for 40 min, 35% to 95% mobile phase B for 1 min, where flow Phase B lasted for 3 min, and 5% mobile phase B was equilibrated for 10 min. The elution peak was monitored at a wavelength of 214 nm, and the component was collected every minute. Components were combined into a total of 10 fractions, which were then freeze-dried.

Date-dependent acquisition (DDA) fractions and Data Independent Acquisition (DIA) sample analysis were performed using the Nano-Liquid Chromatography-Tandem Mass Spectrometry (nanoLC-MS/MS), which was combined with a Q-Exactive HF mass spectrometer (Thermo Fisher Scientific, Waltham, MA, United States) and an Ultimate 3000 RSLCnano system (Thermo Fisher Scientific, Waltham, MA, United States). A nano-LC column (Thermo Fisher Scientific, Waltham, MA, United States) was packed for peptide separation, at a flow rate of 500 nL/min. For DDA analysis, the peptide was loaded into the C18 trap column (Thermo Fisher Scientific, Waltham, MA, United States) with buffer A (2% ACN, 0.1% FA) for 5 min, then eluted gradient with 5 to 25% buffer B (98% ACN, 0.1% FA) for 155 min, 25–30% buffer B for 10 min, and 30–80% buffer B for 5 min. Quantitation analysis for proteomics, including normalization, data transformation, missing imputation, PCA, missing values detection, and differentially expressed proteins (DEPs), was performed using the DEP package (Zhang et al., 2018). The cutoff to define DEPs (differentially expressed proteins) was set as log2| FC| > 1, and the p-adjust was <0.05. Further enrichment analysis was performed as same in RNA-seq.

### Metabolite Profiling

The maize root samples (Y331 and Y331-ΔTE) inoculated with *F. graminearum* were collected at 0 and 24 hpi. Unbiased metabolomic profiles of the maize root samples were performed by using the High Performance Liquid Chromatography-Mass Spectroscopy (HPLC-MS). For each sample, ten biological replicates were used. Around 100 mg of each sample was homogenized in 500 μl of a solvent of acetonitrile-water (7:3) and treated with ultrasonic for 15 min. The mixture was centrifuged at 12,000 rpm at 4°C for 15 min. Prior to liquid chromatographic separation, 200 μl of the supernatant was transferred to a new 1.5 ml polypropylene tube. The LC-MS system operates in a binary gradient solvent mode consisting of 0.1% (v/v) formic acid/water (solvent A) and 0.1% (v/v) formic acid/methanol (solvent B). The C18 columns were used for all subsequent analyses. Sample analysis was carried out in positive electrospray ionization (ESI+) and negative ion (ESI) modes. The column temperature was 45°C with a 5 μl injection volume. The LC-MS data were obtained by using an Agilent 1290 Infinity LC System combined with an Agilent 6530 Accurate-Mass Quadrupole Time-of-Flight (Q-TOF).

For data acquisition, the Agilent MassHunter Workstation QTOF Acquisition software (B.03.01) was used. Raw data files of LC-MS were converted into mzdata format, and data processing was performed by the XCMS toolbox (Tautenhahn et al., 2012). Peak picking was performed by the XCMS software implemented within the R statistical language (v 2.13.1). To identify ion intensities of detected peaks, the retention time (RT)-m/z data pairs were used. The obtained scaled data set was imported into SIMCA-P + 11.0 (Umetrics, Umea, Sweden) for PCA, PLS discriminant analysis (PLS-DA), and orthogonal partial least squares discriminant analysis (OPLS-DA) in order to observe the maximum metabolic changes of each group at all-time points.

Metabolites found to be highly similar were compared to investigate differential alteration in different samples. Differential metabolites were selected when the statistical significance threshold of the variable impact projection (VIP) value obtained from the OPLS-DA model was greater than 1.0 (*p* < 0.05). Based on metabolite abundance, Log2 fold change (FC) was used to show differential metabolites changes. By searching against the online HMDB, METLIN, and KEGG databases with exact molecular mass data from redundant m/z peaks, putative metabolites were determined. Specific metabolites were screened out when the difference between the observed mass and the theoretical mass was less than 10 ppm. To visualize the network of gene ontology terms, Cytoscape 3.8 software with ClueGO plugin was used. For mapping the metabolites and transcript into pathways, Pathview (Version 3.11) was used (Luo et al., 2017).

### Hormone Content Determination by Liquid Chromatography With Tandem Mass Spectrometry (LC-MS/MS)

Fresh maize roots inoculated with *F. graminearum* were harvested at 0, 3, and 6 hpi, immediately frozen in liquid nitrogen and ground into powder, and then stored at –80°C until utilization. Around 50 mg of plant powder sample was dissolved in 1 mL methanol/water/formic acid (15:4:1, V/V/V). For quantification, internal standards were used by adding 10 μL of standard mixed solution (100 ng/mL) into the extract. The mixture was vortexed repeatedly and then centrifuged for 5 min at 4°C (12,000 r/min). The supernatant was transferred for evaporation and dissolved in 100 μL 80% methanol (V/V). Then, the solution was filtered by a 0.22 μm membrane filter for further liquid chromatography with tandem mass spectrometry (LC-MS/MS) analysis using a UPLC-ESI-MS/MS system (UPLC, ExionLC™ AD, AB SCIEX, United States; MS, Applied Biosystems 6500 Triple Quadrupole, Thermo Fisher Scientific, Waltham, MA, United States). The hormone contents were determined by MetWare.^1^

### Quantitative Reverse Transcription PCR

Fresh maize roots inoculated with *F. graminearum* were harvested at 0, 1, 3, and 6 hpi for the test. Total RNA was extracted by using TRIzol reagent (Invitrogen, Carlsbad, CA, United States), and genomic DNA was removed by treating with DNaseI (Invitrogen, Carlsbad, CA, United States). Total RNA sample was used for cDNA synthesis by using a reverse transcription system (Promega, Fitchburg, MA, United States). Then, the quantitative reverse transcription PCR (qRT-PCR) analysis was performed by preparing a reaction system with SYBR Green mix (TAKARA, Dalian, China), and the reaction was running on a real-time PCR detection system (Bio-Rad, California, United States). For each gene (*ZmCCT*, flowering genes, and hormone signaling genes), three replicates were performed. The expression of genes were normalized by comparing with an actin encoding gene *ZmActin1*.

## Results

### Strategy to Investigate Dynamics of Gene Expression Between Y331 and Y331-ΔTE Upon Infection

**FIGURE 1:**
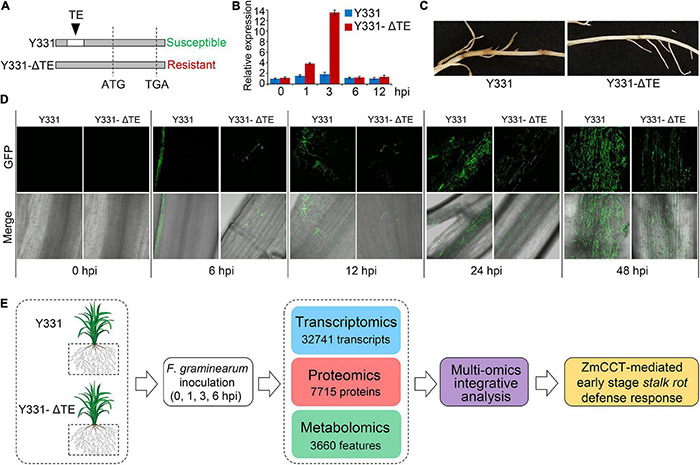
Framework of integrative multi-omic analysis of early-stage defense response to GSR in maize. **(A)** Sketch maps of NILs Y331-ΔTE1 and Y331 at the *ZmCCT* locus. **(B)** Expression profiles of *ZmCCT* in Y331-ΔTE1 and Y331 seedling roots inoculated with *Fusarium graminearum* at different time points. Data were normalized to Y331 at 0 h post-inoculation (hpi). For each sample, the *ZmGAPDH* gene was used as the internal control. Error bars present the ±s.d. of three biological replicates. **(C)** Symptoms of the *F. graminearum*-inoculated maize seedling roots at 72 hpi. **(D)** Growth and distribution of GFP-tagged *F. graminearum* in diseased root tissues of Y331-ΔTE1 and Y331 at different time points. **(E)** Strategy for multiple omics analysis of ZmCCT-mediated defense response in maize.

Previously, a locus encoding *ZmCCT* in maize cultivar Y331 was characterized (Figure 1A; Wang et al., 2017). In Y331, the TE1 element located at the upstream sequence of *ZmCCT* promoter leads to compromised transcription of ZmCCT to promote susceptibility to GSR. The non-TE1 ZmCCT allele in another cultivar Y331-ΔTE is transcriptionally induced at 1–3 hpi and reduced at 6 hpi upon infection and confers resistance to the disease in a transient manner (Figure 1B). These evidence suggest that *ZmCCT* is vital to control resistant response to GSR. First, we tested the pathogenicity of *F. graminearum* against maize cultivars Y331 and Y331-ΔTE (2-week-old seedlings) (Figure 1C), which confirmed that Y331-ΔTE was more resistant than Y331 (Figure 1C). Live-cell imaging using time-course inoculation (at 0, 6, 12, 24, and 48 hpi) by using GFP-labeled *F. graminearum* was observed, and we found that invasive growth of the fungus in the tissue of the susceptible cultivar Y331 was growing more rapidly than that in the resistant cultivar Y331-ΔTE (Figure 1D).

Then, we sought to detect the resistance response by comparing the resistance Y331-ΔTE and susceptible Y331. We deployed the samples of Y331 and Y331-ΔTE inoculated with *F. graminearum* to perform the multi-omic analysis, which were combined with transcriptomic and proteomic analyses at 0, 1, 3, and 6 hpi and metabolomic analysis at 0 and 24 hpi. In total, the analysis collectively characterized 32,741 transcripts, 7715 proteins, and more than 3,660 features as putative metabolite compounds in all the samples (Figure 1E).

### Severe Transcriptional Profiling Changes Between Y331 and Y331-ΔTE Upon Infection

**FIGURE 2:**
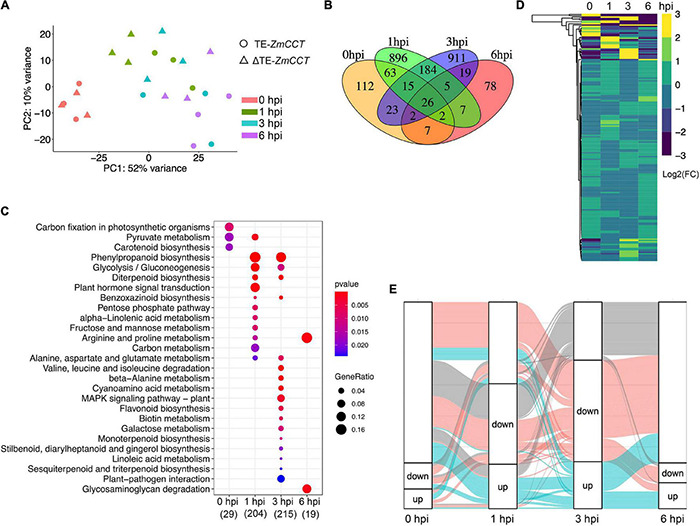
Early-stage resistance to GSR by reprogramming maize transcriptomics. **(A)** The plot showing the principal component analysis (PCA) of maize transcriptomics from Y331-ΔTE and Y331 at four different time points. Normalized reads count was used. **(B)** A Venn diagram showing the numbers of differentially expressed genes (log2| FC| > 1, padj < 0.05) detected in RNA-seq at 0, 1, 3, and 6 hpi. **(C)** The KEGG pathway enrichment analysis using DEGs detected in RNA-seq. **(D)** A heatmap showing relative expression using log2| FC| values from our RNA-seq to demonstrate the expression of ortholog genes between maize and *Arabidopsis*. The *Arabidopsis* RNA-seq data were reported as transcriptionally induced genes responding to fungal elicitor (2021, Nature Plant). **(E)** Alluvial plots of a fraction of a number of differentially expressed genes during infection stages. Each panel shows one time point, and each line represents a different group of DEGs. Red and blue highlight the fractions of DEGs detected at 3 hpi.

To discover the defense-related genes in maize conferring resistant response to GSR during the early stages of infection, we compared the transcriptome signatures crossing four key time points (0, 1, 3, and 6 hpi). After determining the reproducibility by principal component analysis (PCA) (Figure 2A and Supplementary Figure 1B), we compared transcriptional profiling of Y331-ΔTE to Y331 by generating a pairwise comparison for all four time points. We defined 250, 1,198, 1,185, and 146 DEGs (log2| FC| > 1, p-adj < 0.05) at 0, 1, 3, and 6 hpi, respectively (Supplementary Figure 1D and Supplementary Table 1). This result was consistent with the expression pattern of *ZmCCT* in Y331-ΔTE, as the high number of DEGs occurred at 1 and 3 hpi when *ZmCCT* also showed peak expression. Interestingly, the majority of DEGs detected at each time point specifically occurred at each time point. For instance, 74% of DEGs at 1 hpi were not exhibiting significant variation in any other time point and 76% of DEGs at 3 hpi were not showing significant changes in another group of comparisons. Similar patterns were also exhibited at 0 hpi (44%) and 6 hpi (53%) (Figure 2B). This result reflected a rapid reaction triggered as an early response to GSR, and specific variations at 1 and 3 hpi that occurred during defense response would be key factors conferring the resistance.

To further characterize the DEGs, a metabolic pathway enrichment analysis was performed (Supplementary Figure 1), which revealed a number of metabolic pathways potentially associated with defense response to GSR (Supplementary Table 2). For instance, plant hormone signal transduction, pentose phosphate pathway, alpha-linolenic acid metabolism, and fructose and mannose metabolism were specifically enriched at 1 hpi (Figure 2C). DEGs at 3 hpi were specifically enriched in plant-pathogen infection, MAPK signaling pathways, beta-alanine metabolism, biotin metabolism, flavonoid biosynthesis, galactose metabolism, terpenoids biosynthesis, and linoleic acid metabolism (Tang et al., 2021). By connecting multiple metabolic pathways enriched in DEGs, we observed glycolysis/gluconeogenesis, phenylpropanoid biosynthesis, diterpenoid biosynthesis, benzoxazinoid biosynthesis, and alanine, aspartate, and glutamate metabolisms were co-regulated by DEGs at 1 and 3 hpi, indicating that these pathways were probably important to activate defense response in maize at the early stage of infection (Figure 2C). Strikingly, only two metabolisms, arginine and proline metabolisms and glycosaminoglycan degradation, were enriched at 6 hpi, suggesting that ZmCCT-associated resistance to GSR is an early response during infection before 6 hpi. Metabolism of carbon fixation in photosynthetic organisms and pyruvate metabolism were enriched at 0 hpi, suggesting ZmCCT functions in photosynthesis before fungal infection. Gene ontology (GO) enrichment analysis yielded a number of functions such as peroxidase activity and plant-type cell wall, and the response to oxidative stress are enriched in DEGs at 1 hpi. We also discovered that DEGs at 3 hpi were enriched in biological processes associated with nucleosome, MCM complex, nuclear nucleosome, and plasma membrane (Supplementary Figure 1 and Supplementary Table 3).

The top differentially expressed genes were shown in Supplementary Figure 2. To validate the DEGs results, we selected seven differentially expressed genes to perform qRT-PCR experiment, which showed a high consistency between RNA-seq and qRT-PCR results (Supplementary Figure 2). To get more insights into underlying key properties conferring GSR resistance, we compared the DEGs in our RNA-seq to PTI-induced genes in *Arabidopsis* (Bjornson et al., 2021). In total, 933 maize DEGs were orthologous genes of *Arabidopsis* PTI-responsive genes (Figure 2D and Supplementary Table 4). Many of these genes were involved in pathways reported as key properties in PTI, such as the MAPK signaling pathway, phenylpropanoid biosynthesis, and glycolysis pathways (Supplementary Figure 3). At 3 hpi, we observed the highest number of DEGs during early resistance to GSR. By visualizing the fractions of DEGs at different time points, we observed that a big proportion of DEGs at 3 hpi were also transactionally induced or repressed at 1 hpi, many of which were not transcriptionally induced at 6 hpi (Figure 2E). This result demonstrated a set of genes that specifically altered their transcriptional levels upon infection and triggered a transient resistance to stalk rot at 1–3 hpi.

### Weighted Correlation Network Analysis Defined Nine Modules as Transcriptional Co-expressed Maize Genes Responding to Gibberella Stalk Rot

**FIGURE 3:**
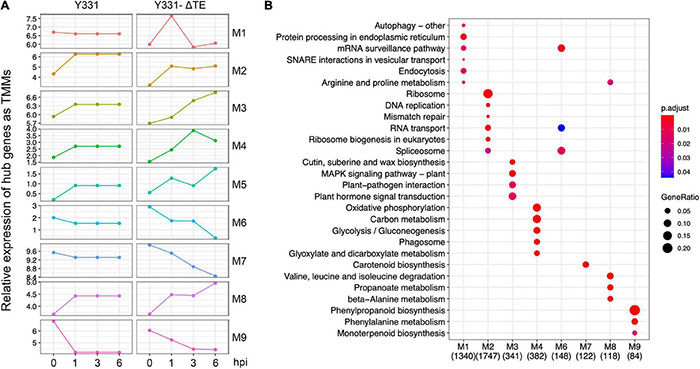
Weighted correlation network analysis (WGCNA) analysis defined nine co-expressed genes as modules during infection stages. **(A)** The plots showing relative expression as TPM values of hub genes representing nine coexpressed genes modules derived from WGCNA analysis using all samples. **(B)** A dot plot showing the significance of KEGG pathways enriched by each coexpression module.

To uncover more insights into the underlying resistance mechanism of maize to GSR, we performed WGCNA analysis using all the samples at four time points. Nine different modules were identified by clustering analysis (Supplementary Table 5). To elucidate the expression pattern of these modules of co-expressed genes, we visualized the expression of hub genes, which are the representative ones in each module (Figure 3A), and performed metabolic pathway enrichment analysis to determine their functions (Figure 3B). This co-expression analysis provided a landscape of dynamic gene expression to illustrate transcriptional divergence in resistant and susceptible maize. In module 1, 7105 genes were transcriptionally co-expressed, which represented about 15% of all genes in the genome of maize. These genes exhibited peak expression at 1 hpi in the resistant cultivar Y331-ΔTE, but a flat pattern in the susceptible cultivar Y331. This result suggests that these genes were transcriptionally induced as a response to GSR. Enrichment analysis indicates that these genes were tightly associated with pathways of the autophagy process, vesicular transport, and endocytosis to facilitate resistant response, and maize modulates a series of pathways associated with secretion and the autophagy process (Figure 3B). Similarly, in modules 3, 4, 5, and 8, genes exhibited peak expression at 3 hpi and/or 6 hpi in Y331-ΔTE (Figure 3A), suggesting that these genes were co-expressed and induced at these time points to mediate the resistance. These genes were significantly enriched in metabolic pathways of the MAPK signaling pathway and plant hormone signal transduction, which is reported as core components to confer PTI (Figure 3B). By contrast, in modules 2, 6, 7, and 9, gene expression shows a reduced pattern in Y331-ΔTE samples at 1, 3, and 6 hpi, indicating that these genes are transcriptionally repressed at these stages. Taken together, the co-expression network analysis demonstrated a sophisticated transcriptomics circuit reprogrammed in Y331-ΔTE and revealed the autophagy process and secretion pathways, MAPK pathways, and plant hormone pathways that were vital in the early-stage response to GSR.

### ZmCCT Coordinates Different Cellular Processes for Resistance to Stalk Rot Disease in Maize

**FIGURE 4:**
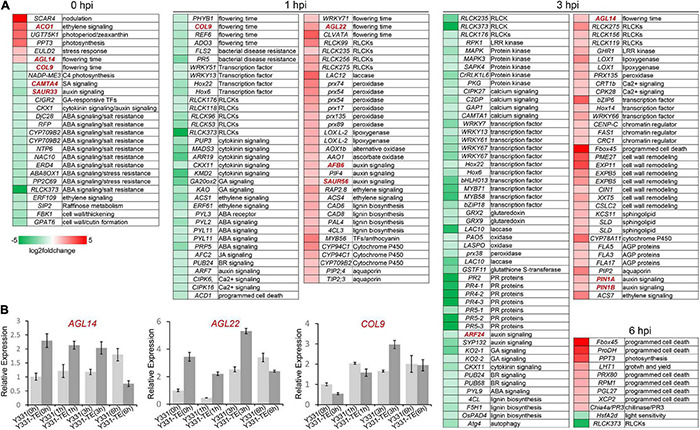
Mapping of differentially expressed genes in maize during different infection stages of *Fusarium graminearum.* **(A)** The heatmaps showed detailed relative expression (log2 fold change) of selected genes related to important functions from RNA-seq. Genes were named according to their *Arabidopsis* or rice orthologs. **(B)** Relative expression of several putative flowering time-related genes in *F. graminearum*-infected maize seedling root at different time points. The β-actin gene *ZmActin* was used as an internal control for normalization. For each gene, the relative enrichment value in Y331-infected seedling root cells at 0 hpi was assigned as 1. Error bars present the ±s.d. of three biological replicates.

To widen the analysis and interpret more insights into the resistance mechanism in maize to GSR, we sought to investigate the orthologous genes with known functions by extracting orthologous genes in maize to *Arabidopsis* and rice. Using the RNAseq analysis, DEGs were identified, and using proteomics analysis, DEPs were identified, and the known function of the orthologous genes was identified by mining the published data and reports in *Arabidopsis* and rice genes (Supplementary Table 6 and Figure 4A).

To explore why ZmCCT can coordinate photoperiod and defense response, the known function of genes associated with the photoperiod process were identified. Interestingly, we found four genes significantly induced by *F. graminearum* infection at 1 or 3 hpi are known as key factors regulating flowering development in *Arabidopsis*, *AGL14*, *AGL22*, *WRKY71*, and *CLVATA* (Figure 4A). In contrast, several other *Arabidopsis* homologous genes, CONSTANS-like gene *COL9*, phytochrome gene *PHYB1*, histone H3 lysine 27 demethylase gene *REF6* were severely repressed during *F. graminearum* infection at 1 or 3 hpi (Figure 4A). The MADS transcription factor AGL14 modulates auxin transport during *Arabidopsis* root development by regulating the expression of *PIN* genes (Garay-Arroyo et al., 2013). Overexpression of *AGL14* in *Arabidopsis* results in early flowering, suggesting that it also regulates flowering time (Pérez-Ruiz et al., 2015). Another MADS transcription factor, AGL22, is involved in the transition from vegetative state to the flowering stage (Bechtold et al., 2016), and deletion of *AGL22* resulted in early flowering similar to the phenotype of non-functional ZmCCT (Y331) (Gregis et al., 2013). Transcription factor WRKY71 accelerates flowering via the direct activation of the flowering time integrator gene FLOWERING LOCUS T and the floral meristem identity genes LEAFY (Yu et al., 2016). The CLAVATA gene is also reported to modulate flowering time and flower number in chickpeas (Basu et al., 2019). We also identified several genes homologous to *A. thaliana* CONSTANS-like gene *COL9*, phytochrome gene *PHYB1*, histone H3 lysine 27 demethylase gene *REF6* (a relative of early flowering 6), and PAS domain gene *ADO3*, which were severely repressed during *F. graminearum* infection at 1 or 3 hpi (Figure 4A). *COL9* delays the flowering time by repressing the Ehd1 pathway in *Oryza sativa* and *A. thaliana* (Cheng and Wang, 2005; Liu H. et al., 2016). *REF6* encodes a Jumonji N/C and zinc finger domain-containing protein that acts as a positive regulator of flowering in an FLC-dependent pathway (Cui et al., 2016). The qRT-PCR experiment confirmed that expression patterns of selected flowering-related genes, *AGL14*, *AGL22*, and *COL9* were consistent with the omics data (Figure 4B). Altogether, these data suggested that ZmCCT may be involved in coordinating photoperiod and defense response through key regulatory genes related to flowering.

At 1 and 3 hpi, many known functions of homologous genes of DEGs/DEPs were reported to be involved in PTI response, including genes encoding RLCK kinases (RLCK99, RLCK156, RLCK119, RLCK235, RLCK256, RLCK275, and GHR1), redox proteins (laccase, peroxidase, lipoxygenase, and oxidase), hormone pathway proteins, transcription regulators (WRKY66, Hox14, bZIP6, CENP-C, FAS1, and CRC1), aquaporins, AGPs proteins, lignin biosynthesis proteins, and sphingolipid biosynthesis proteins (Figure 4A). Interestingly, expressions of some redox-related genes, protein kinases, calcium signaling pathway proteins, the lignin biosynthesis genes, and several disease resistance proteins (FLS2, PR2, PR4, PR5) were significantly repressed at 3 hpi, suggesting a weaker PTI response at 3 hpi and a different cellular process comparing to that at 1 hpi.

When inoculated onto susceptible maize cultivar, *F. graminearum* undergoes a biotrophic growth stage at least before 12 hpi (Zhang et al., 2016). Interestingly, some programmed cell death-related genes, including genes homologous to *A. thaliana* ubiquitin E3 ligase component *Fbox45*, proline dehydrogenase *ProDH*, peroxidase *PRX80*, *PGL27*, and xylem cysteine protease *XCP2* (Avci et al., 2008), as well as an R gene *RPM1* (Boyes et al., 1998), were significantly upregulated in Y331-ΔTE at 6 hpi in resistant maize (Figure 4A). These data suggested that the Y331-ΔTE resistant line launches a hypersensitive reaction strategy for resistance at 6 hpi. To test this possibility, we stained the *F. graminearum*-infected root seedling cells with Trypan blue, which was commonly used to stain dead cells in plants. At 0, 1, and 3 hpi, none of the *F. graminearum*-infected host cells could be stained to deep blue color, while at 6 hpi, the infected host cells were much easier to be stained to deep blue in Y331-ΔTE but not in Y331 (Supplementary Figure 4), suggesting that cell death occurred, which is consistent with the prediction.

### Different Hormone Pathways Were Coordinated for Early-Stage Resistance to Gibberella Stalk Rot

Plant defense against microbial attack is regulated by a complex network of hormone signaling pathways (Robert-Seilaniantz et al., 2011). We identified that a large number of DEGs/DEPs were involved in hormone pathways during maize responding to GSR. At 0 hpi before *F. graminearum* infection, many genes involved in hormone signaling pathways were repressed in resistant line Y331-ΔTE (Figure 4A). Genes encoding auxin receptor F-box protein AFB6 (Prigge et al., 2016), bHLH transcription factor PIF4 (Choi and Oh, 2016), and small auxin upregulated protein SAUR56 were activated at 1 hpi, while the other two genes encoding *PIN1* (*PIN1A* and *PIN1B*) were activated at 3 hpi (Figure 4A). Several other auxin signaling pathway genes, auxin response transcription factor genes, *ARF7*, *ARF24*, and syntaxin *SYP132* (Xia et al., 2020) were decreased in Y331-ΔTE at 1 or 3 hpi.

**FIGURE 5:**
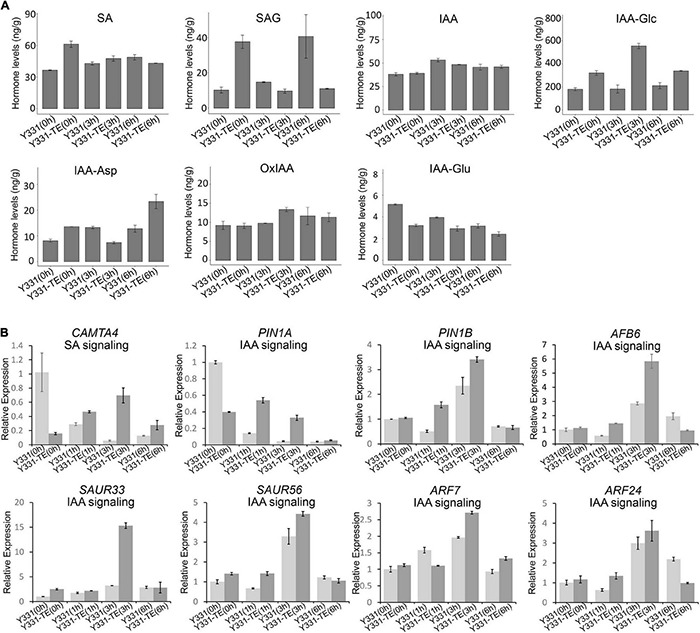
Hormone level changes in Y331-ΔTE and Y331 during infection of *Fusarium graminearum.* **(A)** Determination of endogenous maize hormone contents by LC-MS/MS during *F. graminearum* infection on Y331-ΔTE or Y331 at different time points. SA, salicylic acid; SAG, salicylic acid 2-O-β-glucoside; IAA, indole-3-acetic acid; IAA-Asp, indole-3-acetyl-L-aspartic acid; IAA-Glc, 1-O-indol-3-ylacetylglucose; OxIAA, 2-oxindole-3-acetic acid; IAA-Glu, indole-3-acetyl glutamic acid. **(B)** Relative expression of hormone signaling pathway-related genes in *F. graminearum*-infected maize seedling root at different time points. The β-actin gene *ZmActin* was used as an internal control for normalization. For each gene, the relative enrichment value in Y331-infected seedling root cells at 0 hpi was assigned as 1. Error bars present the ±s.d. of three biological replicates.

We measured the contents of chemical compounds related to SA and IAA hormones in both Y331 and Y331-ΔTE during *F. graminearum* infection. In non-inoculated roots, the SA level was higher in Y331-ΔTE than in Y331, but there was no evident difference between these two lines upon fungal infection at 3 and 6 hpi (Figure 5A). In addition to the regulation at the biosynthesis level, free SA also undergoes chemical modifications, including glycosylation, to form the inactive SA-glucoside (SAG) (Lefevere et al., 2020). At 0 hpi, SAG was also higher in Y331-ΔTE but decreased in both Y331-ΔTE and Y331 at 3 hpi (Figure 5A). Interestingly, it was much higher in Y331 at 6 hpi, suggesting that SA could massively exist as an inactive form in Y331 at this time point. We evaluated the expression pattern of *CAMTA4* by qRT-PCR. As a putative repressor of SA biosynthesis, *CAMTA4* was significantly decreased in Y331-ΔTE under the non-treatment condition; coincidently, the SA level was elevated when compared with that in Y331. Upon *F. graminearum* infection at 1 and 3 hpi, the expression level of *CAMTA4* in Y331-ΔTE was increased when compared with that in Y331 but reduced to the level under the non-treatment condition, which was correspondent with the SA signaling pathway (Figure 5B).

In general, the IAA levels were slightly elevated during *F. graminearum* infection, but no evident differences were observed between the resistant line Y331-ΔTE and the susceptible line Y331 at 3 and 6 hpi (Figure 5A). As a major storage form of auxin in plants, auxin conjugates also play a key role in regulating the availability of endogenous-free IAA (Ludwig-Müller, 2011). The mainly detected auxin conjugates in root samples of Y331-ΔTE and Y331 include IAA-Glc, IAA-Asp, OxIAA, and IAA-Glu, among which the content of IAA-Glc accounted for the vast majority (Figure 5A). Interestingly, the IAA-Glc level was significantly increased in Y331-ΔTE compared with that in Y331 at each detecting time, suggesting a possible role of ZmCCT in the regulation of IAA-Glc to affect IAA signaling pathway during *F. graminearum* infection. We chose several IAA signaling pathway genes mentioned above to confirm their expression pattern during *F. graminearum* infection (Figure 4A). As expected, most of these genes, including *PIN1A*, *PIN1B*, *AFB6*, SAUR33, and SAUR56, ARF7 and ARF24, were activated at 1 or 3 hpi but reduced to the level similar to that of non-treatment condition (Figure 5B). These results showed that these IAA signaling pathway genes were affected at the early stage of GSR.

**FIGURE 6:**
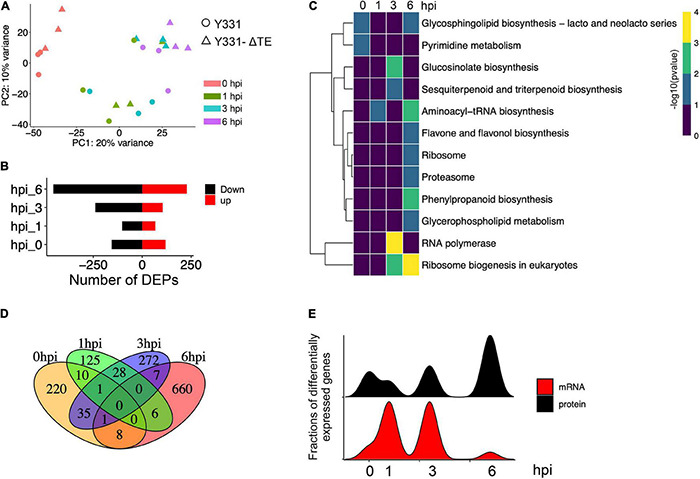
Proteomic analysis revealed a stronger response to infection at the translational level at 6 hpi. **(A)** Principal component analysis of maize proteomics from Y331-ΔTE and Y331 at different time points. **(B)** Barplot showing numbers of DEPs as upregulated and downregulated proteins. **(C)** A dot plot showing the significance of KEGG pathways enriched by DEPs detected in proteomics. **(D)** A Venn diagram showing numbers of differentially expressed proteins when comparing Y331-ΔTE and Y331 at 0, 1 3, and 6 hpi after inoculation. **(E)** Density plot showing the relative fraction of DEGs in RNA-seq, and DEPs in proteomics at four time points.

After data normalization, quality control, and the PCA (Figure 6A and Supplementary Figure 5), comparative proteomic analysis identified 275, 170, 344, and 682 proteins as differentially expressed proteins (DEPs) at 0, 1, 3, and 6 hpi, respectively (Figure 6B and Supplementary Table 7). These results indicated a rapid increase of proteins at 6 hpi during defense response to GSR (Supplementary Figure 6). The GO enrichment analysis indicated that DEPs at 6 hpi were significantly enriched in terms associated with nucleic acid binding, RNA binding, RNA helicase activity, nuclear transport, and rRNA process (Supplementary Figure 6). This result suggested a specific function of DEPs at 6 hpi in RNA process and binding, which may also continue to modulate defense response at 6 hpi (Supplementary Figures 7A,B and Supplementary Table 8). We discovered these DEPs are significantly enriched in metabolic pathways in glucosinolate biosynthesis at 3 hpi and phenylpropanoid biosynthesis at 6 hpi (Figure 6C and Supplementary Table 9). Similar to the result of DEGs, DEPs at four time points were not well-overlapped (Figure 6D), suggesting that, at each time point during the early stage, the expression of the specific group of genes were significantly altered at both transcriptional and translational levels. To further integrate RNA-seq and proteomic data, we generated correlation analysis and found an uncorrelated pattern in samples at all four time points, suggesting that the abundance of transcripts and proteins were not equal during defense response (Supplementary Figure 7). By comparing the variation patterns of RNA-seq and proteomic analyses, we found that the defense response triggers transcriptional reprogramming at 1 and 3 hpi, but the translational level was most changed at 6 hpi (Figure 6E).

**FIGURE 7:**
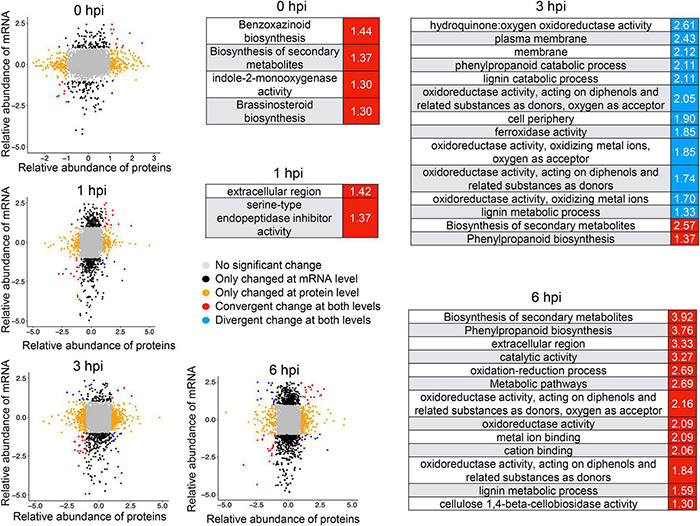
Integrative analysis of RNA-seq and proteomics identified a number of genes co-induced upon resistance to stalk rot disease. The mRNA/protein expression fold changes of samples were calculated by comparing Y331-ΔTE and Y331 at four time points. mRNAs/proteins differentially expressed (| log2[FC]| > 1; *p*-adj < 0.05) in both, either and neither (unchanged) transcriptome and proteome studies were grouped and color-coded. Red and blue represent the Gene Ontology term significantly enriched by co-induced or co-repressed genes at transcriptional and translational levels, respectively.

Integrative analysis of RNA-seq and proteomics data also defined a set of genes induced at both transcriptional and translational levels (Figure 7), among which genes involved in brassinosteroid biosynthesis and benzoxazinmoid biosynthesis were enriched at 0 hpi and genes involved in the phenylpropanoid biosynthesis pathway were enriched at 3 hpi (Figure 7). There were more co-induced genes at 6 hpi, which were significantly enriched in phenylpropanoid biosynthesis and the oxidation–reduction process (Figure 7), suggesting that these biological processes and pathways might be vital to conferring resistance to GSR. These results highlighted a set of important genes that show convergent patterns at mRNA and protein levels. At the same time, some genes showing divergent patterns at mRNA and protein levels at four time points were also detected (Figure 7). Notably, the divergent pattern genes at 3 hpi were significantly enriched in many GO terms, including the lignin catabolic process, cell periphery, and plasma membrane (Figure 7), suggesting that a turnover regulatory event occurred in these genes.

**FIGURE 8:**
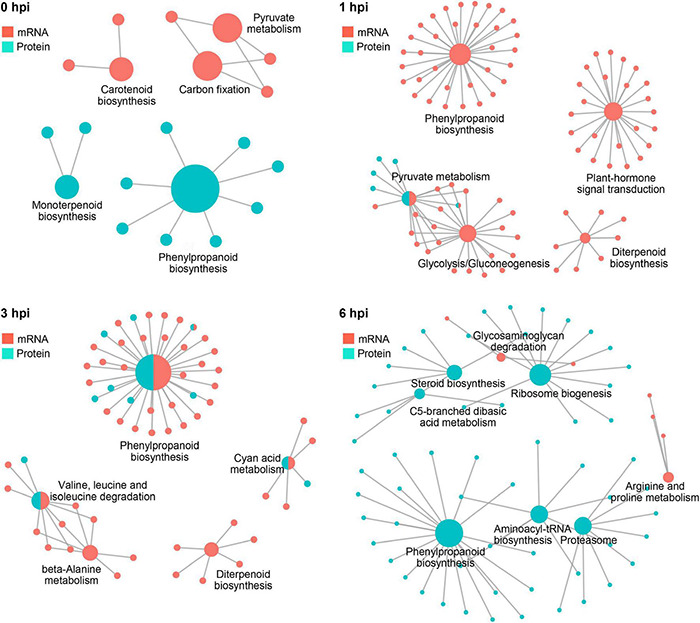
The complex association between enriched KEGG pathways. Plots showing KEGG pathways significantly enriched by DEGs and DEPs detected in RNA-seq and proteomics at 0, 1, 3, and 6 hpi.

Further integrative analysis with metabolic pathways demonstrated a clear dominant regulatory effect in the mRNA level at 1 hpi and in the protein level at 6 hpi (Figure 8), suggesting a transient response to stalk rot at a very early stage in the transcriptional level after 1 hpi and in the translational level at 6 hpi. Taken together, the combination of RNA-seq and proteomic analyses demonstrated a distinct responsive pattern in transcriptional and translational levels during early defense response to GSR in maize.

### Genome-Wide Metabolomics Analysis Uncovered Key Properties Associated With Defense to Gibberella Stalk Rot

**FIGURE 9:**
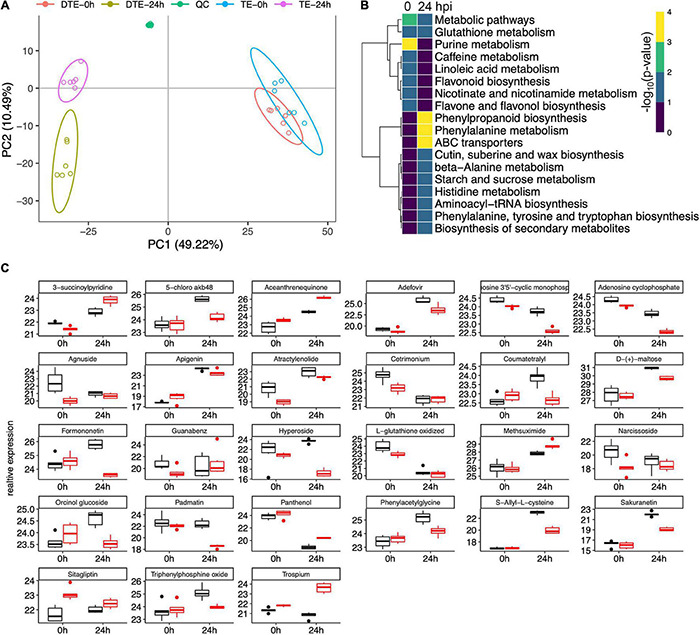
Metabolomic analysis revealed key pathways associated with early-stage resistance to gibberella stalk rot (GSR). **(A)** Principal component analysis to determine the global metabolic variation between Y331-ΔTE and Y331 at 0 and 24 hpi. **(B)** Heatmap showing the significance of metabolic pathways enriched by metabolic compounds detected in comparative metabolomics comparing Y331-ΔTE and Y331. **(C)** Boxplots showing the relative abundance of detected metabolic compounds in comparative metabolomics which are significantly altered between Y331-ΔTE and Y331. Black refers to Y331, and red refers to Y331-TE.

Given that a sophisticated gene expression reprogramming was affected during the resistance to GSR at the early stages of infection, we set up to explore the outcome of these regulatory events. Using genome-wide metabolomics to compare Y331-ΔTE and Y331 samples, after inoculation at 0 and 24 hpi, we profiled 3,660 putative features from all the samples after normalization (Supplementary Figure 8). The PCA and clustering analysis demonstrated a distinct metabolomic pattern between Y331-ΔTE and Y331 at 24 hpi (Figure 9A). A significantly distinct metabolomic pattern between 0 and 24 hpi was also observed (Figure 9A), suggesting a massive metabolic outcome of transcriptional regulation.

Differentially expressed compounds analysis defined 8 and 12 enriched metabolic pathways at 0 and 24 hpi, respectively (Figure 9B, Supplementary Figure 9, and Supplementary Tables 10, 11). At 24 hpi, the phenylpropanoid biosynthesis, phenylalanine metabolism, and ABC transporters were severely increased (Figure 9B), suggesting key roles of these metabolic processes in GSR defense response. Several other metabolic processes, including cutin, suberin, and wax biosynthesis, beta-alanine metabolism, starch and sucrose metabolism, histidine metabolism, aminoacyl-tRNA biosynthesis, phenylalanine, tyrosine, and tryptophan biosynthesis, as well as biosynthesis of secondary metabolites, were also enriched (Figure 9B), suggesting that these metabolic processes were also involved in GSR defense response. Figure 9C showed a series of detected metabolic compounds in comparative metabolomics which were significantly altered between Y331-ΔTE and Y331.

**FIGURE 10:**
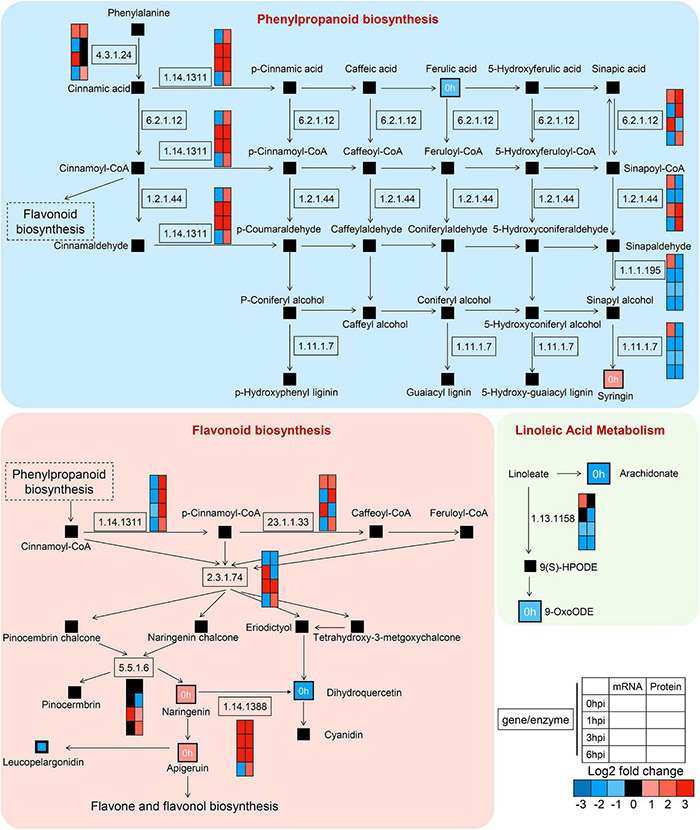
High-resolution mapping of the regulatory network to metabolic pathways. Simplified metabolic flow schemes described changes in metabolites and enriched enzymes associated with transcripts, proteins, phenylpropanoid biosynthesis, flavonoid biosynthesis, and linoleic acid metabolism. The colors indicated rates of upregulation or downregulation of groups of enzymes.

To demonstrate the regulatory network during early GSR defense response in maize, we mapped the DEGs, DEPs, and altered compounds in phenylpropanoid biosynthesis and flavonoid biosynthesis, which were known to play key roles in plant defense (Figure 10). We found severe upregulation in genes at the transcriptional level relevant to enzymatic reactions, such as 23.1.1.133 in flavonoid biosynthesis and 1.14.1311 in phenylpropanoid biosynthesis (Figure 10). We detected a concurrent upregulated abundance of gene products at both transcriptional and translational levels in genes associated with enzymatic reaction 1.14.1388 at 0, 1, and 3 hpi, which negatively regulates the abundance of dihydroquercetin. Interestingly, we found that, in the linoleic acid metabolism, genes and proteins associated with enzymatic reaction 1.13.1158 at 1, 3, and 6 hpi were all decreased, suggesting that this metabolic process was negatively associated with ZmCCT upon infection. Taken together, these results demonstrated detailed information on the metabolic processes in maize’s early GSR defense.

## Discussion

The filamentous fungus *F. graminearum* infects maize as a hemibiotrophic lifestyle to cause GSR. As a consequence of arm racing, maize has evolved complex defense strategies to encounter fungal infection at different cellular levels (Kazan et al., 2012). Given that the maize quantitative resistance gene *ZmCCT* is transcriptionally induced in maize at the early infection stage of *F. graminearum* (1–3 hpi) for defense, it can be used as a key resource to study the regulatory mechanism of maize’s early-stage defense response to GSR. We found that, at the early infection stage of *F. graminearum*, maize rapidly launches a massive PTI-mediated defense response pattern. Transcriptional profiling indicates a transit reaction that is served at 1 and 3 hpi. Comparing the gene expression variation patterns in RNA-seq and proteomics, we found that, in Y331-ΔTE, defense triggers transcriptional reprogram at 1 and 3 hpi but a severe change occurs at the translational level at 6 hpi. Combining with the metabolomic analysis, we discovered some key metabolic pathways that might be important for resistance response to GSR. Our study also suggested that *ZmCCT* fine-tunes the cellular processes between defense and photoperiod response. Altogether, the study produces a great resource to interpret the early defense mechanism during GSR resistance in maize.

### Multi-Omic Strategy Provides a Comprehensive Understanding of Gibberella Stalk Rot Resistance

The multi-omic analysis has been used to explore the mechanisms of plant disease resistance (Crandall et al., 2020), which provides more in-depth insights into the molecular mechanism of defense response to the infection of the pathogens. For example, the roles of ZmHIR3 in maize resistance to GSR were revealed by integrative analyses of gene co-expression analysis and metabolites profiling (Sun et al., 2021). Besides, transcriptomic and metabolomic integrative analyses of the resistant and susceptible lines of Fusarium head blight caused by *F. gramineae* was also carried out, which identified that several enzymes and transcription factors were candidate resistance genes (Dhokane et al., 2016). A comparative proteomic analysis revealed ZmWRKY83-mediated GSR resistance (Bai et al., 2021). Combined with DAP-Seq and RNA-Seq analyses, a recent study also showed the regulatory mechanism of ZmCCT in coordinating flowering, stress response, and development (Su et al., 2021). In this study, we combined transcriptomic data with those proteomic and metabolomic approaches to comprehensively reveal a multilevel landscape of maize defense response and better understand the mechanism of GSR resistance at the early biotrophic growth stage during *F. graminearum* infection.

### The Resistance Mechanism of Gibberella Stalk Rot in the Early Stage of Infection Is Closely Related to Pattern-Trigged Immunity

*Fusarium graminearum* possesses a hemibiotrophic lifestyle upon the infection of maize which causes GSR (Glazebrook, 2005; Brown et al., 2010; Kazan et al., 2012). At the early infection stage, *F. graminearum* remains non-symptomatic for a period before the development of a necrotrophic phase (Brown et al., 2010; Kazan et al., 2012). To encounter *F. graminearum* infection, maize have also developed complex defense strategies at both transcriptional and translational levels. Considering that the lifestyles of *F. graminearum* during different phases of disease development are quite different, correspondingly, the host plant also evolves different resistance responses during different infection stages. Previous studies have shown that various plant defense signaling pathways show spatio-temporal dynamics and stage-specific patterns in response to pathogen attacks (Ding et al., 2011; Zhang X. W. et al., 2012; Brown et al., 2017). For example, Ca^2+^ signaling pathway and SA signaling pathway were usually induced at the early infection stage, but JA signaling pathway was usually induced at the late infection stage (Ding et al., 2011). A transcriptomic analysis comparing lines with and without qRfg1 showed that defense-associated genes are quickly induced at the early infection stage of GSR (Yang et al., 2010; Ye et al., 2013; Liu Y. et al., 2016). In this study, the integrative transcriptomic, proteomic, and metabolomic analyses clearly showed the different patterns of DEGs, DEPs, and DAMs, further supporting that early-stage GSR resistance is strongly activated at the early infection stage of *F. graminearum*.

To provide a wider vision of transcriptional profiling associated with GSR resistance in the early stage, we deployed WGCNA to construct co-expression networks, in which a series of genes were identified to be significantly enriched at the early stage of fungal infection, including genes associated with pathways of autophagy process, vesicular transport, and endocytosis. Genes involved in metabolic pathways of MAPK signaling pathway, plant hormone signal transduction, glycolysis, and phagosome were significantly enriched in modules co-expressed and induced at 3 hpi and/or 6 hpi to mediate the resistant (Figure 3). More interestingly, most of these genes are reported as core components to confer the PTI-mediated defense Response (Supplementary Figure 3). Our result is consistent with a previous study in barley, which showed that the transcriptome analysis of barley revealed that *F. graminearum* infection-induced differential expression of genes was related to the PTI-mediated defense response (Bigeard et al., 2015). Future studies should focus on dissecting the function and molecular mechanisms, including the PTI-related defense response underlying the GSR resistance.

### ZmCCT Coordinates Defense and Photoperiod at the Early-Stage Response to Gibberella Stalk Rot

Previous studies have shown that the qRfg1 locus not only increases maize resistance to GSR by activating the expression of defense-related genes but also fine-tunes the metabolic processes to balance defense and growth. Our multi-omic analysis provides a more detailed explanation for important defense-related biological processes in the resistant line Y331-ΔTE (Figures 2, 3). We found in maize that many orthologous rice or *Arabidopsis* genes were reported to be involved in flavonoid, phytoalexin, redox, RLCKs, and WRKY transcription factors, which are also detected as DEGs in our analysis. These results suggest that Y331-ΔTE can counterattack the infection of *F. graminearum* via constitutive resistance.

Pathogen infection activates phytohormone signaling pathways which, in turn, mediates signaling transmission to trigger plant immunity (Bari and Jones, 2009). However, pathogens have evolved effectors as secreted proteins to interfere with plant hormone signaling pathways (Han and Kahmann, 2019). Genes upregulated in Y331-ΔTE is an action as a response to the SA signaling pathway and auxin signaling pathway that is believed to be associated with FHB resistance (Hao et al., 2018), suggesting the role of *ZmCCT* in the constitutive resistance to infection by regulating phytohormones. The *ZmCCT* gene seems to contribute to GSR resistance by positively influencing the expression of auxin signaling pathway genes (Figure 4). Experimental detection confirmed the elevation of auxin signaling during *F. graminearum* infection in the resistant line Y331-ΔTE. As discussed above, *ZmCCT* functions at the early biotrophic stage; therefore, we speculate that, during GSR resistance, the auxin signaling pathway is affected by ZmCCT for early biotrophic stage resistance and repressed by other regulators (such as ZmHIR3) for late necrotrophic stage resistance. Recently, a transcriptome and oxylipin profiling joint analysis indicated that 9-oxylipins contribute to resistance but JAs facilitate susceptibility during GSR (Wang et al., 2021). Another recent study showed that ZmCOIa and endogenous JA may function as susceptibility factors during GSR (Ma et al., 2021).

*ZmCCT* is one of the most important genes regulating photoperiod response. The presence of *ZmCCT* blocked the flowering transition of maize seedlings, which was characterized by increased plant height and delayed flowering (Yang et al., 2010). There may be an internal relationship between flowering time and resistance because late flowering is usually related to strong disease resistance (Elzinga et al., 2007). It was found that *ZmCCT* plays its role in a tissue-specific pattern, which shows strong photoperiod sensitivity in leaves, but conferred stable defense response to GSR in roots (Wang et al., 2017). A chromatin remodeling of the *ZmCCT* promoter may be a major epigenetic factor in the regulation of *ZmCCT* expression. It seems reasonable for plants to use such a chromatin-based regulatory mechanism to orchestrate basal and stress-induced gene regulation in a precise and timely manner to balance the trade-offs of growth and pathogen defense. Here, we identified a series of flowering controlling genes that were activated or repressed in Y331-ΔTE, among which, *AGL14*, *AGL22*, and *COL9* were confirmed by the qRT-PCR analysis, suggesting that *ZmCCT* can well balance the flowering-defense balance by coordinating downstream signaling networks. It is interesting to reveal the regulatory mechanisms between *ZmCCT* and these identified flowering controlling genes in the future.

In summary, this study clearly shows that the multi-omic analysis has been very useful in advancing our overall understanding of maize defense response to GSR, which significantly advanced our understanding of this economically important plant–microbe interaction.

## Data Availability Statement

The original contributions presented in the study are publicly available. This data can be found here: NCBI, PRJNA757397 and National Genomics Data Center, PRJCA006316.

## Author Contributions

WW, X-LC, and BT designed the project. ZZ, XZ, YX, and LW performed the experiments. BT and X-LC performed the multi-omic data analysis and interpretation of the results. X-LC, BT, ZZ, and WW wrote the manuscript. All authors contributed to the article and approved the submitted version.

## Conflict of Interest

The authors declare that the research was conducted in the absence of any commercial or financial relationships that could be construed as a potential conflict of interest.

## Publisher’s Note

All claims expressed in this article are solely those of the authors and do not necessarily represent those of their affiliated organizations, or those of the publisher, the editors and the reviewers. Any product that may be evaluated in this article, or claim that may be made by its manufacturer, is not guaranteed or endorsed by the publisher.

## Footnotes

1
http://www.metware.cn/
