# Supplementary material for: Multi-Omics Analysis Reveals a Regulatory Network of ZmCCT During Maize Resistance to Gibberella Stalk Rot at the Early Stage
Source: Front Plant Sci. 2022 Jun 23;13:917493. doi: 10.3389/fpls.2022.917493 (PMC9260664; doi:10.3389/fpls.2022.917493)
Supplement: Supplementary file 12 [file Data_Sheet_1.pdf]

Supplementary Figures S1-S9

Multi-omics analysis reveals a regulatory network of ZmCCT-mediated maize resistance to Gibberella stalk rot at the early stage

Bozeng Tang[1†], Zhaoheng Zhang[1†], Xinyu Zhao[1], Yang Xu[1], Li Wang[1], Xiao-Lin Chen[2*] and Weixiang Wang[1*]

[1] Beijing Key Laboratory of New Technology in Agricultural Application, National Demonstration Center for Experimental Plant Production Education, College of Plant Science and Technology, Beijing University of Agriculture, Beijing, China.

[2] State Key Laboratory of Agricultural Microbiology and Provincial Hubei Key Laboratory of Plant Pathology, College of Plant Science and Technology, Huazhong Agricultural University, Wuhan, China.

[†]These authors contributed equally to the work.

[*]Correspondence: wwxbua@163.com or chenxiaolin@mail.hzau.edu.cn

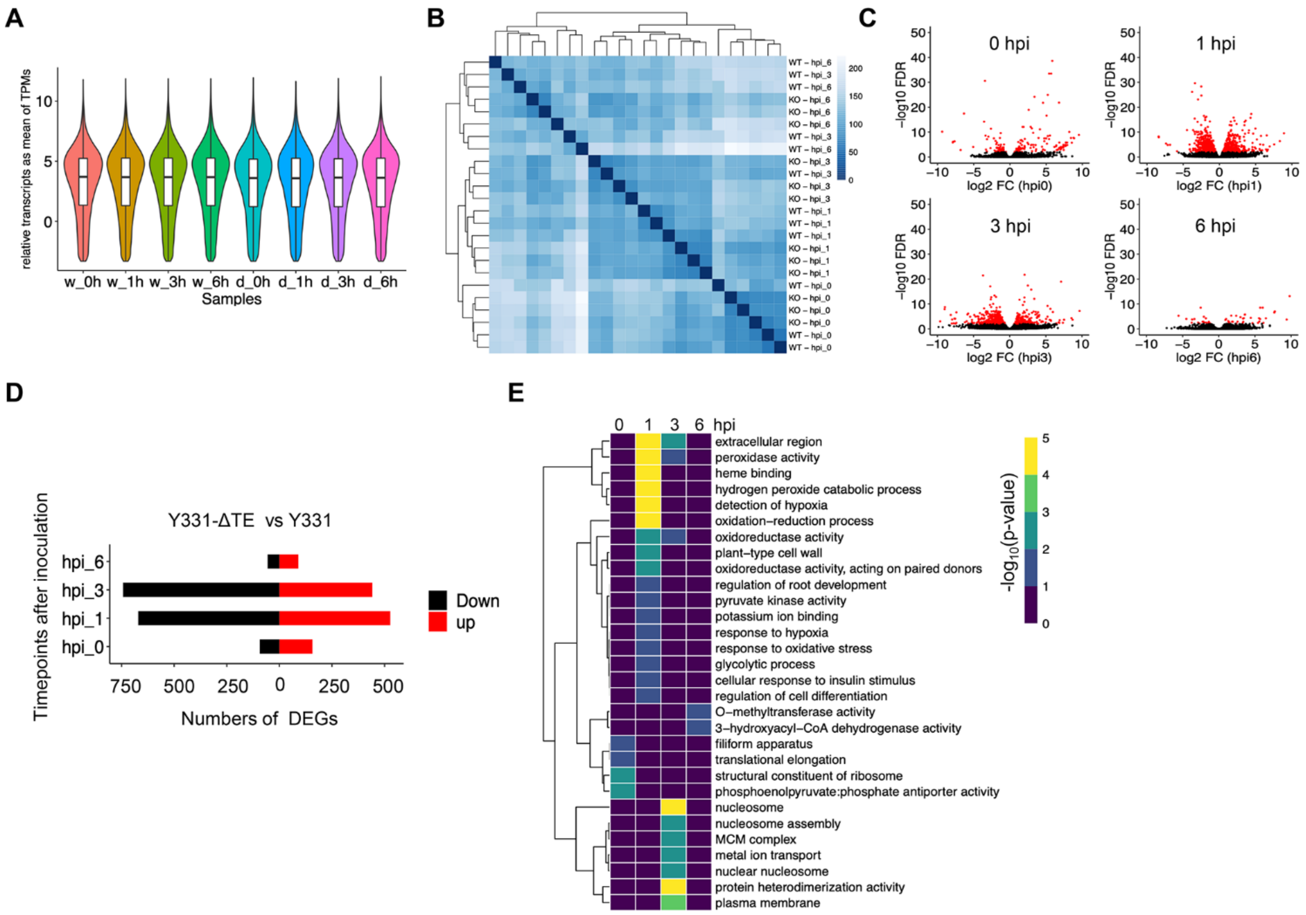

Fig. S1. Comparative transcriptomics to study resistant response to stalk rot disease associated with ZmCCT. (A) Violin-plot showing distributions of TPMs as normalized gene expression detected in RNA-seq of different samples from Y331-ΔTE and Y331. (B) Heatmap showing result of clustering analysis to determine the similarity between samples of RNA-seq. (C) Volcano-plot showing the result of differentially expressed genes identification. X-axis represent log2|FC| values, and Y-axis represent -log10 |FDR| at 0hpi, 1hpi, 3hpi, and 6hpi. The red dots represent DEGs after filtering (FC > 1, adj < 0.05). (D) Barplot showing the numbers of upregulated (red) and down-regulated genes detected at four time points in RNA-seq after comparing Y331-ΔTE and Y331. (E) Heatmap showing the result of Gene Ontology enrichment analysis using DEGs identified in RNA-seq analysis. The values above -log10 (p-value) are considered as significantly enriched.

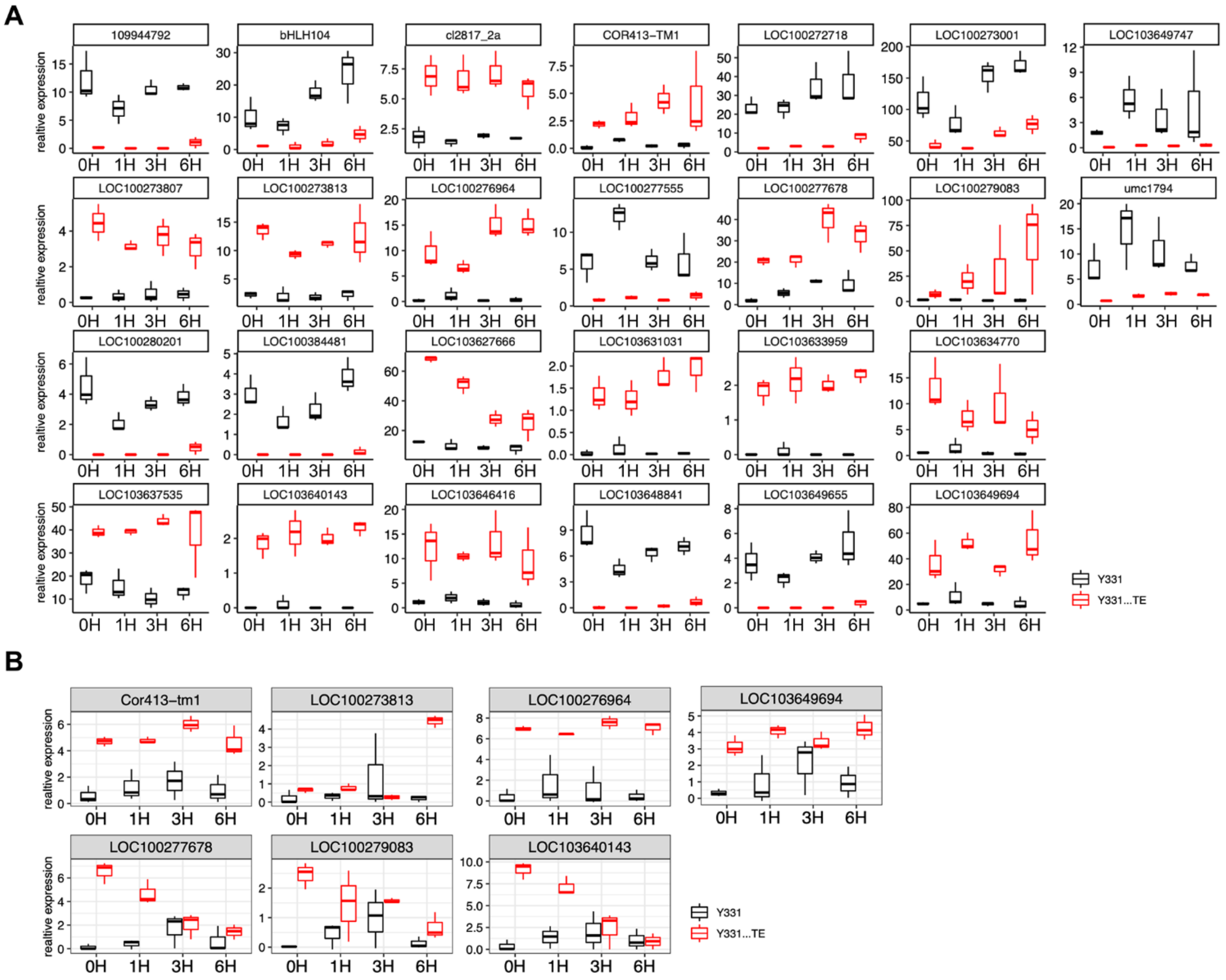

Fig. S2. A set of genes transcriptionally induced during response to stalk rot. (A) Boxplots showing the normalized values of gene expression detected in RNA-seq at four timepoints for top 26 genes selected from DEGs for each time point. (B) Boxplot showing the relative transcripts of 7 genes detected in qRT-PCR. For each sample, the *ZmGAPDH* gene was used as internal control. Three replicates are performed for each experiment.

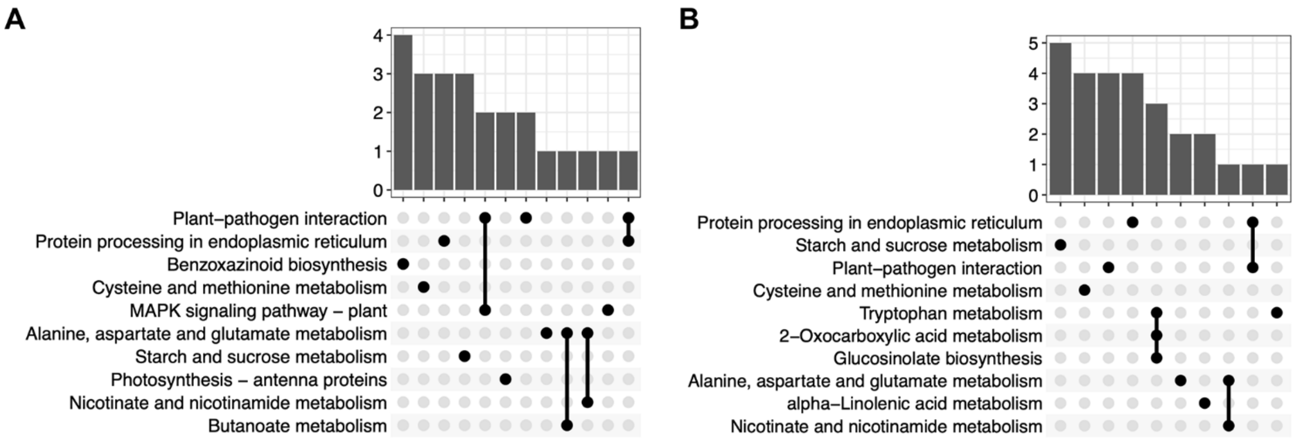

Fig. S3. Annotations of maize genes as orthologs to Arabidopsis induced by PTI. (A) The plot showing detailed annotations of maize genes as orthologs to Arabidopsis, which has been reported to be transcriptionally induced in PTI (Bjornson, et al., 2021). The result is showing distribution of the gene set in maize metabolic pathway. (B) The plot showing detailed annotations of Arabidopsis, which has been reported to be transcriptionally induced in PTI (Bjornson, et al., 2021), but also as DEGs in this study. The result is showing distribution of the gene set in Arabidopsis metabolic pathway.

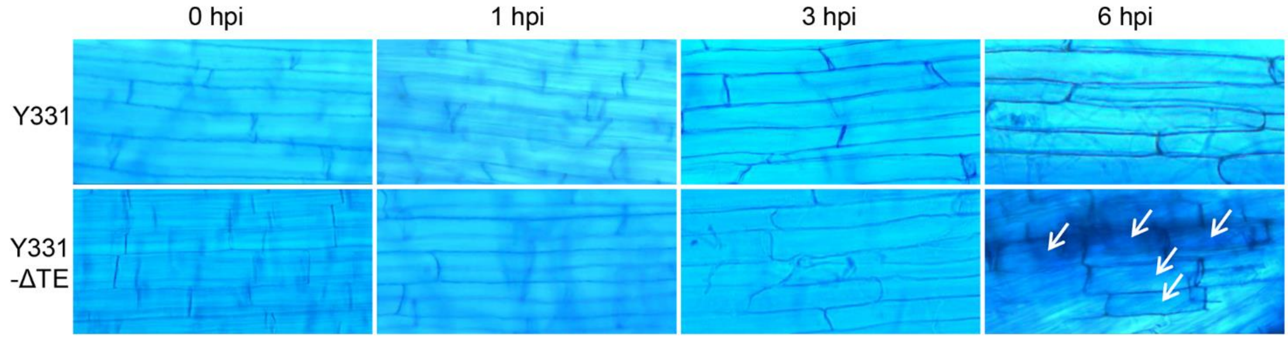

Fig. S4. Trypan blue staining assay showed cell death of *F. graminearum*-infected maize seedling root cells at different time points after inoculation. White arrow indicates the death cell which can be stained by Trypan blue.

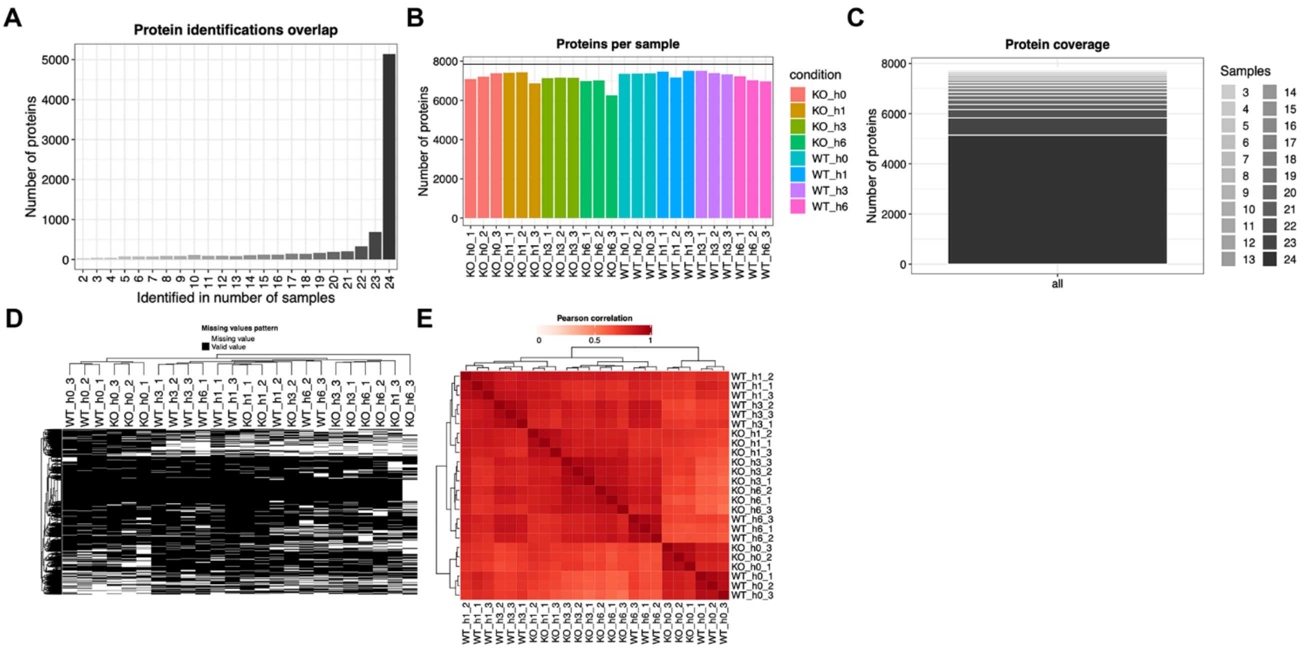

Fig. S5. Quality control and normalization of proteomics to study resistant response to stalk rot associated with ZmCCT. (A) Numbers of proteins detected from all 24 proteomics datasets. (B) Detailed numbers of detected proteins after alignment against maize amino acid sequence in all samples. KO represent the samples of Y331-ΔTE, and WT represent samples of Y331. (C) Coverage of the proteomics datasets cross all 24 samples. (D) The heatmap showing present and absent of detected proteins in proteomics. (E) Similarity and distance between all 24 samples in proteomics. Pearson analysis was performed to determine the correlation between each pair of samples.

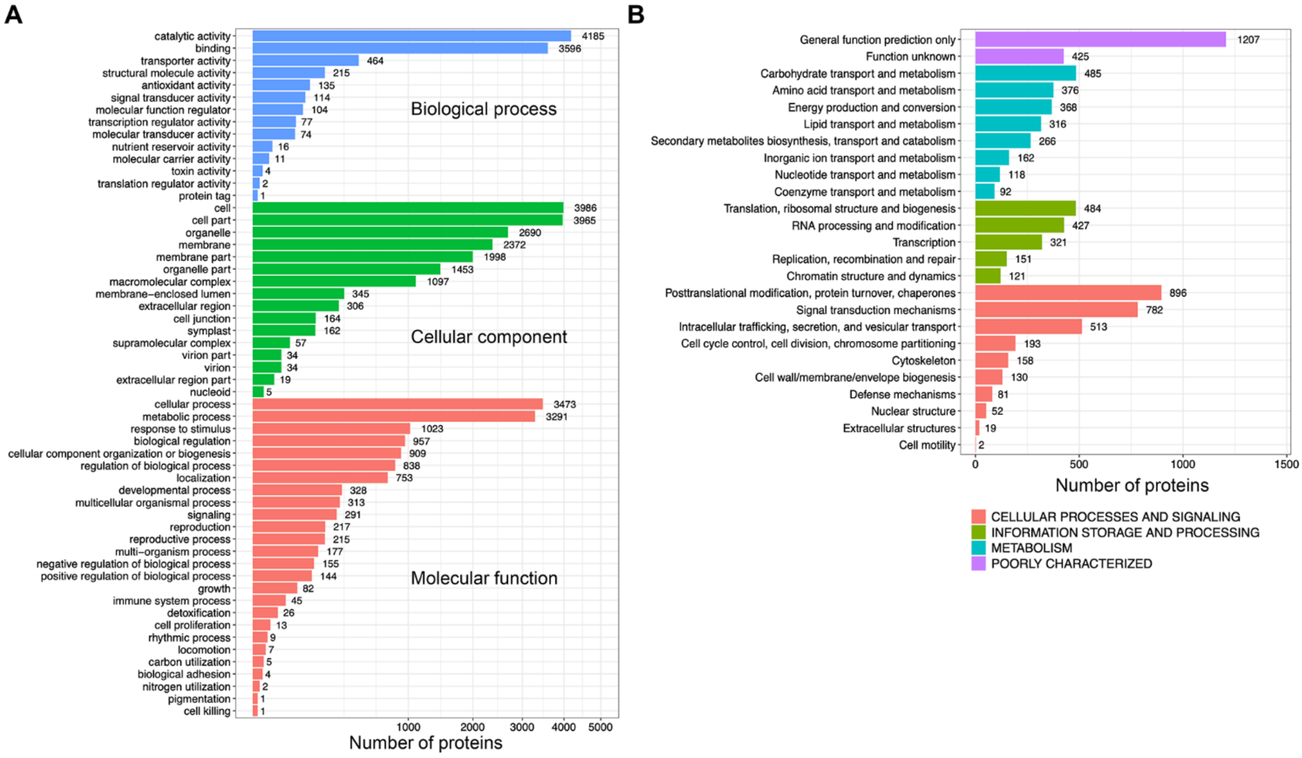

Fig. S6. Diverse biological processes, molecular functions, and biological components are annotated by the proteins detected in proteomics analysis. (A) Numbers of proteins in each category as Gene Ontology term, as biological process, cellular component, and molecular functions, from all 24 samples in proteomics. (B) Numbers of proteins in each category as Gene Ontology term, as biology functions, from all 24 samples in proteomics.

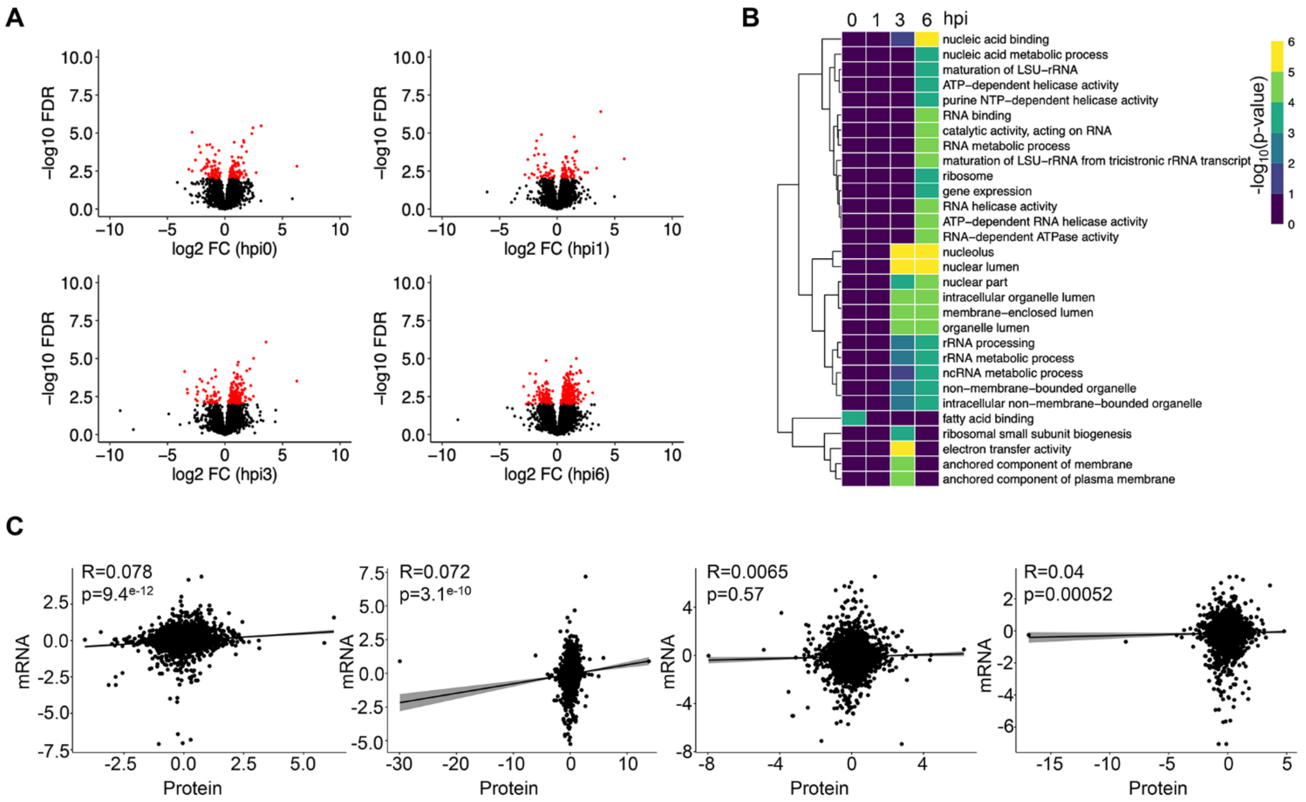

Fig. S7. Differentially expressed proteins analysis. (A) Volcano-plot showing the result of differentially expressed proteins (DEPs) identification in proteomics analysis. X-axis represent log2|FC| values, and Y-axis represent -log10 |FDR| at 0 hpi, 1 hpi, 3 hpi, and 6 hpi. The red dots represent DEPs after filtering (FC > 1, adj < 0.05). (B) The heatmap showing the result of Gene Ontology enrichment analysis using DEPs identified in proteomics analysis. The values above -log10 (p-value) are considered as significantly enriched. (C) Pearson corelation analysis to determine relative changes of mRNA and proteins in maize at 0 hpi, 1 hpi, 3 hpi, and 6 hpi. The x-axis indicates fold changes values obtained for protein, and y-axis indicates fold changes values obtained for mRNA. The R values indicate the degree of correlation.

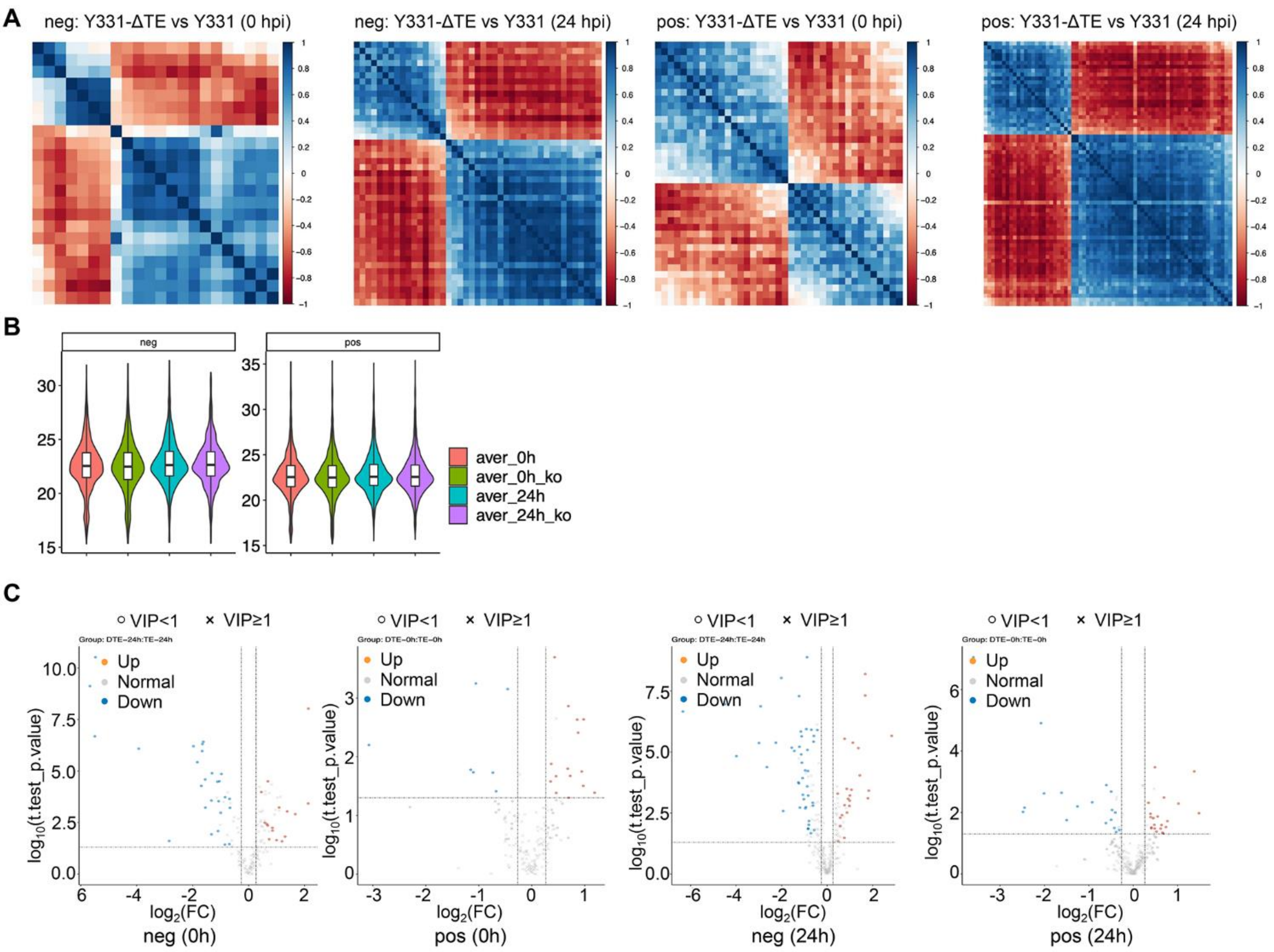

Fig. S8. Normalization and comparison analysis in metabolomics. (A) Heatmaps showing the correlations between detected features in metabolomics at 0 hpi and 24 hpi from neg and pos polarity MS. (B) Violin plot showing distributions of averaged normalized abundance of features detected in metabolome analysis between Y331-ΔTE and Y331. (C) Volcano-plots showing the differentially expressed features as putative metabolic compounds detected in neg and pos polarity metabolome dataset between Y331-ΔTE and Y331 of maize with inoculation by *F. graminearum* at 0 hpi and 24 hpi.

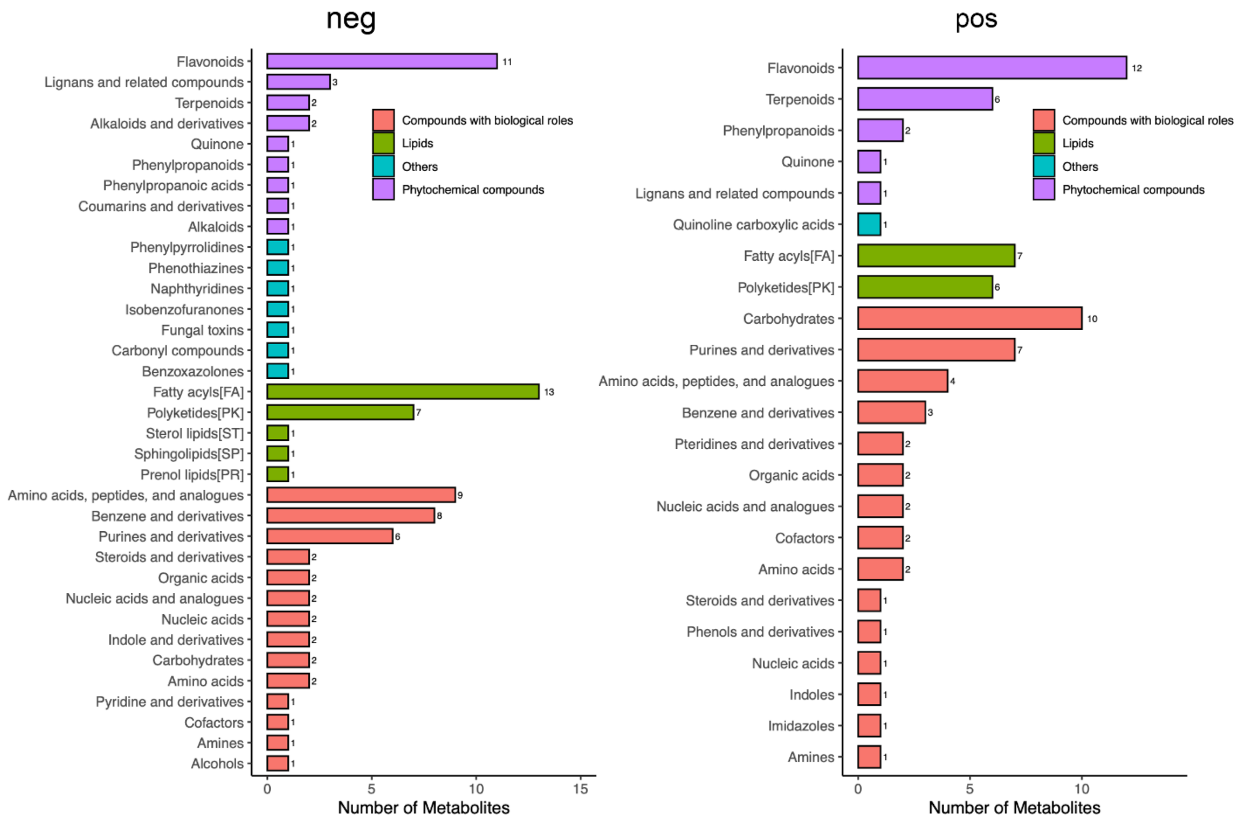

Fig. S9. Annotations of putative metabolic compounds detected in metabolomics. X-axis represent the numbers of each class of compounds, and y-axis represent the classes. Neg, negative; pos, positive.
